# Genetic Diversity of Food-Isolated *Salmonella* Strains through Pulsed Field Gel Electrophoresis (PFGE) and Enterobacterial Repetitive Intergenic Consensus (ERIC-PCR)

**DOI:** 10.1371/journal.pone.0081315

**Published:** 2013-12-03

**Authors:** Imen Fendri, Amal Ben Hassena, Noel Grosset, Mohamed Barkallah, Lamia Khannous, Victoria Chuat, Michel Gautier, Radhouane Gdoura

**Affiliations:** 1 Unité de recherche Toxicologie - Microbiologie Environnementale et Santé, Faculté des Sciences de Sfax, Université de Sfax, Sfax, Tunisia; 2 Laboratoire de Microbiologie, Département agroalimentaire Agrocampus Ouest, Rennes, France; 3 INRA, CIRMBIA, UMR 1253 Science et technologie du lait et de l'œuf (STLO), Agrocampus ouest, Rennes, France; U. S. Salinity Lab, United States of America

## Abstract

All over the world, the incidence of *Salmonella* spp contamination on different food sources like broilers, clams and cow milk has increased rapidly in recent years. The multifaceted properties of *Salomnella* serovars allow the microorganism to grow and multiply in various food matrices, even under adverse conditions. Therefore, methods are needed to detect and trace this pathogen along the entire food supply network. In the present work, PFGE and ERIC-PCR were used to subtype 45 *Salmonella* isolates belonging to different serovars and derived from different food origins. Among these isolates, *S.* Enteritidis and *S.* Kentucky were found to be the most predominant serovars. The Discrimination Index obtained by ERIC-PCR (0.85) was slightly below the acceptable confidence value. The best discriminatory ability was observed when PFGE typing method was used alone (DI = 0.94) or combined with ERIC-PCR (DI = 0.93). A wide variety of profiles was observed between the different serovars using PFGE or/and ERIC-PCR. This diversity is particularly important when the sample origins are varied and even within the same sampling origin.

## Introduction

Salmonellosis is a major health problem worldwide and accounts for high morbidity rates. Infection with *Salmonella enterica* occurs mainly through the consumption of contaminated food, and the estimated annual number of human infections is greater than 93.8 million cases, with 155,000 deaths per year worldwide [1]. Many *Salmonella* serovar infections result in diarrheal diseases, bacteraemia and extraintestinal focal infections in infants and more serious complications among the elderly and immunocompromised adults [2]. The pathogenicity [3] and the increase of antimicrobial resistance in *Salmonella* have been recognized as the ultimate causes. Filter feeding organisms such as clams harvested from contaminated waters are known to concentrate high levels of *Salmonella* serovars leading to a high incidence of this pathogen on seafood [4]. This is also the case in broilers [5]. However, information on milk contamination is scarce.

Different phenotypic and biochemical characteristics have been previously used for the epidemiological investigation of *Salmonella* [6], [7]. Beyond the phenotypic characterization, a reliable genetic level discriminatory method is required. In fact, molecular typing methods that rely on DNA sequence differences are essential for the epidemiological study of pathogenic *Salmonella* serovars [8]. Bacterial housekeeping genes were widely used for molecular typing and were based on polymorphisms analysis in defined genetic loci in the bacterial genome by the PCR amplification and sequencing of the PCR products [9].

Randomly Amplified Polymorphic DNA (RAPD) and Enterobacterial Repetitive Intergenic Consensus (ERIC) fingerprinting were recently used to differentiate *Salmonella* serovars in seafood and human origins [10]–[12]. An earlier study compared four molecular typing methods for the differentiation of *Salmonella spp* (RAPD, ERIC, Ribotyping PCR and Single Strand Conformation Polymorphism (SSCP)) and observed ERIC-PCR to be the most efficient [11]. ERIC is a short interspersed repetitive consensus sequence originally found in *E*.*coli* and *Salmonella* and ERIC-PCR uses outward facing primers complementary to each end of the repeat in a PCR [13]. On the other hand, the application of pulsed-field gel electrophoresis (PFGE) has been proved to be useful for the discrimination and epidemiological characterization of *Salmonella enterica* strains [14]–[16].

In the present study, the serotyped *Salmonella* isolated from Tunisian clams, broilers and milk were subjected to DNA based fingerprinting using PFGE and ERIC-PCR in order to characterize *Salmonella* isolates collected from different origins and to define the relationships between them.

## Materials and Methods

### *Salmonella* isolation and serotyping

All the samples were analyzed according to the International Organization for Standardization Method 6579 (ISO). Isolation and biochemical identification were carried out according to standard laboratory methods. Suspected *Salmonella* colonies were screened using real time PCR. 1 ml of isolates culture was centrifuged at 13,000 g for 20 min. The pellet was resuspended in 200 µl sterile water. The total volume was extracted by Quick-gDNA MiniPrep D3006 Kit (Zymo Research,CA, USA) as recommended by the manufacturer. Extracted DNA was re-suspended in 50 µl of elution buffer and stored at −20°C until subsequent analysis [17]. Real-time PCR was performed on the CFX96™ real-time PCR cycler (Biorad). Amplification reactions were carried out at a final volume of 25 µl containing 0.2 µM of each primer (Table 1), 12.5 µl of 2× SYBR® Permix Ex Taq™ Tli RNaseH Plus (TaKaRa) and 1 µl of genomic DNA. PCR amplification was conducted by incubating the samples at 95 C for 3 s, followed by 40 cycles of 5 s at 95°c and 30 s at 60°C. Primers amplifying *invA* gene were used as previously described [18]. A single confirmed *Salmonella* isolate from each positive sample was serotyped according to the Kauffman–White scheme using commercial antisera [19]. Serotyping was carried out at the National Centre of Enteropathogenic Bacteria, Pasteur Institute, Tunis.

**Table 1:** Distribution of *Salmonella* isolates derived from broilers, clams and cow milk.

*Salmonella* serovars	Origin	Number of strains	Total	Frequency (%)
Enteritidis	Broiler Intestine	5	19	42.2
	Broiler Liver	7		
	Broiler Carcass	5		
	Clam	1		
Kentucky	Broiler Intestine	6	18	40.0
	Broiler Liver	4		
	Cow Milk	8		
Anatum	Cow Milk	2	2	4.4
London	Clam	2	2	4.4
Irenea	Clam	2	2	4.4
Poona	Clam	1	1	2.2
Brancaster	Clam	1	1	2.2

### *Salmonella* isolates selection

Forty five *S*. *enterica* isolates were selected for further molecular typing. These isolates were obtained from i) a total tissue of 7 clams from the sampling station M2 located at the Golf of Gabes (Southern Mediterranean). Sampling was carried out manually by randomly picking up clams off the coast. The sampling process was supervised by the Commissariat Régional du Développement Agricole de Mednine (CRDA). ii) 28 samples derived from intestine, carcass and liver of sexed chickens (HubbardJV) collected at a meat processing industry situated in the region of Sfax and iii) 10 samples of cow milk collected from farms (Sfax, Tunisia) after the consent of their owners. Permission to use these animal parts was obtained from the slaughterhouse (Sfax, Tunisia) to use these animal parts. The origin of each isolate and its appropriated serotype are given in Table 1.

### Pulsed-field gel electrophoresis (PFGE)

Pulsed-field gel electrophoresis was preformed according to the one-day (24 to 28 h) standardized laboratory protocol for the molecular subtyping of *Salmonella* by PFGE (Pulse- Net, Centers for Disease Control and Prevention, Atlanta, Ga.), (Centers for Disease Control and Prevention, 2005), and as described by Ribot et al. [20] with minor changes. Cells were suspended in a wash buffer (10 mM Tris-HCl pH 7.6, 1 M NaCl) and then lysed in a lysis buffer (10 mM Tris-HCl, 750 mM EDTA pH 9, 10% of N-Lauroylsarcosine (Sigma Aldrich, France) and 14 mg/ml proteinase K (Eurobio, France). Genomic DNA was prepared by embedding *Salmonella* isolates cells in agarose plugs (Invitrogen, France). XbaI (New England Biolabs, UK) was used to digest the DNA for each isolate. Electrophoresis was performed in a Biorad Chef-DR®-II system in 0.5X Tris-Borate-EDTA (TBE) extended-range buffer (Eurobio, France) with recirculation at 14°C. The following settings were used for DNA migration: Step I with an initial switch time of 20 sec, a final switch time of 45 sec, a gradient of 6 V/cm and 9 h of electrophoresis; Step II with initial switch time 5 sec, final switch time 15 sec, a gradient of 6 V/cm and 10 h of electrophoresis. Three lambda markers (lambda DNA cI857 ind 1 Sam7, GelSyringe™, New England Biolabs, UK) were included on each gel. Following electrophoresis, the gel was stained with Gel-Red 3X in a 0.1 M NaCl solution (FluoProber, Interchim), visualized under UV light and then photographed.

### ERIC-PCR for *Salmonella* isolates

For ERIC-PCR, the primers ERIC-1R (5′-ATGTAAGCTCCTGGGGATTCAC-3′) and ERIC2 (5′AAGTAAGTGACTGGGGTGAGCG-3′) (Sigma Aldrich) [13], [21] were used with some changes. The PCR was performed in a 50 µL solution containing 1 µM of each primer, 5 µL of 10X PCR buffer, 250 µM dNTPs, 3 mM MgCl_2_, and 3.0 U of Taq DNA polymerase (Sigma Aldrich). The PCR conditions were one cycle at 95°C for 10 min, followed by 4 cycles of 5 min at 94°C, 5 min at 40°C and 5 min at 72°C and then followed by 30 cycles of 1 min at 94°C, 1 min at 55°C, and 2 min at 72°C, and the last extension at 72°C for 10 min. A 10 µl aliquot of each amplification reaction was analyzed using electrophoresis on a 2% agarose gel and run in a 1X TBE buffer, pH 8.3. The gel was stained with Gel-Red 3X in a 0.1 M NaCl solution (FluoProber, Interchim) and photographed. A 1000 base pair Smart Ladder (Eurogentec, France) was included on the gel as a marker.

### Data Analysis

The banding patterns from PFGE and ERIC-PCR analysis were analyzed with BioNumerics Software version 6.5 (Applied-Maths, Ghent, Belgium). The similarities between strains were calculated using the Dice coefficient with an optimization of 1%. The dendrograms were obtained by means of the Unweighted Pair Group Method with Arithmetic Average (UPMGA) clustering algorithm. Numerical index of discriminatory ability of PFGE, ERIC-PCR and combined typing methods were calculated by applying Simpson's Index of Diversity equation as previously described by Nath et al. [12].

### Ethical statement

None of the authors of this paper has a financial or personal relationship with other people or organizations that could inappropriately influence or bias the content of this work.

## Results

### Serotyping of *Salmonella* isolates

In this work, 45 *Salmonella enterica* isolates were recovered from different origins (Table 1). After serotyping, nine distinct serovars were identified, among which two were dominant: *Salmonella* Enteritidis (n = 19) and *Salmonella* Kentucky (n = 18). Minor serovars included *Salmonella* Anatum (n = 2), *Salmonella* Irenea (n = 2) and *Salmonella* London (n = 2). Only one isolate was recovered for each of *S*. Poona and *S*. Brancaster (Table 1).

### Verification of *Salmonella* isolates by real time PCR

All isolated strains were serotyped as *Salmonella* strains. To confirm this, all strains were analyzed by real time PCR using primers previously described as *invA* gene specific [22]. PCR results obtained in this study indicated that all *Salmonella* strains tested by PCR were positive for the presence of a 284 bp fragment of the *invA* gene.

### PFGE typing results

**Figure 1:**
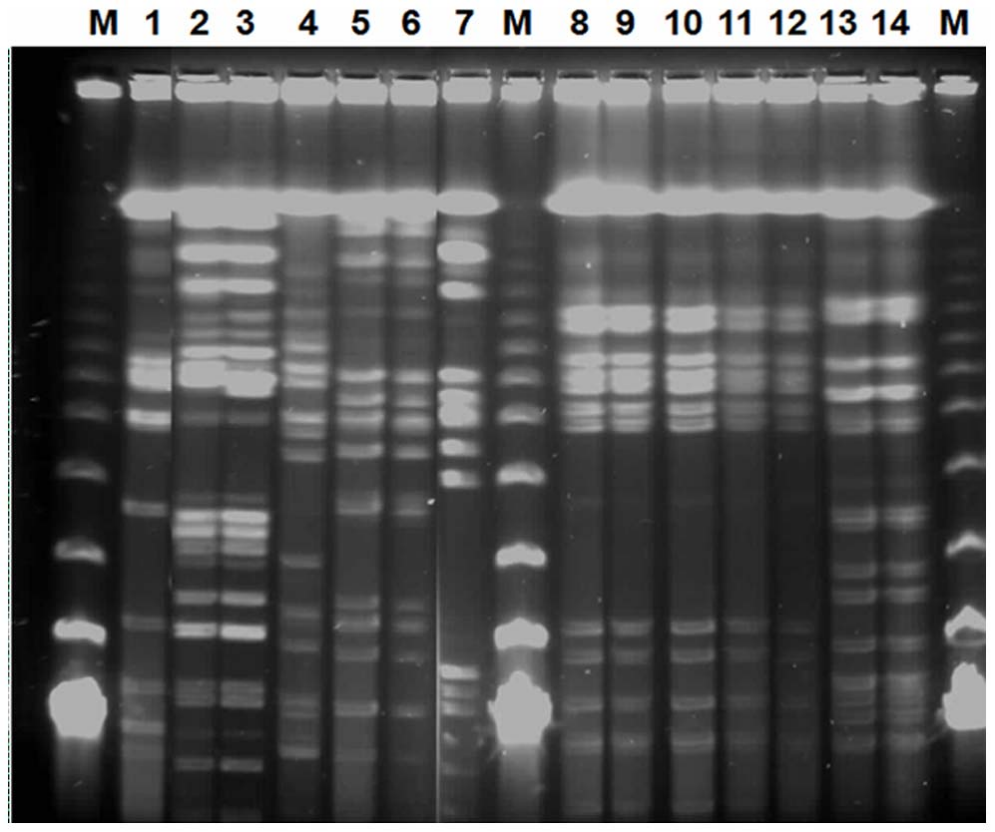
Representative PFGE fingerprint of differences between *Salmonella* isolates on 1 per cent agarose gel. M, lambda DNA marker; lane 1 to 14: O, P, Q, K, L, M, T, L1, L2, L3, L4, L5, L9 and L10.

**Figure 2:**
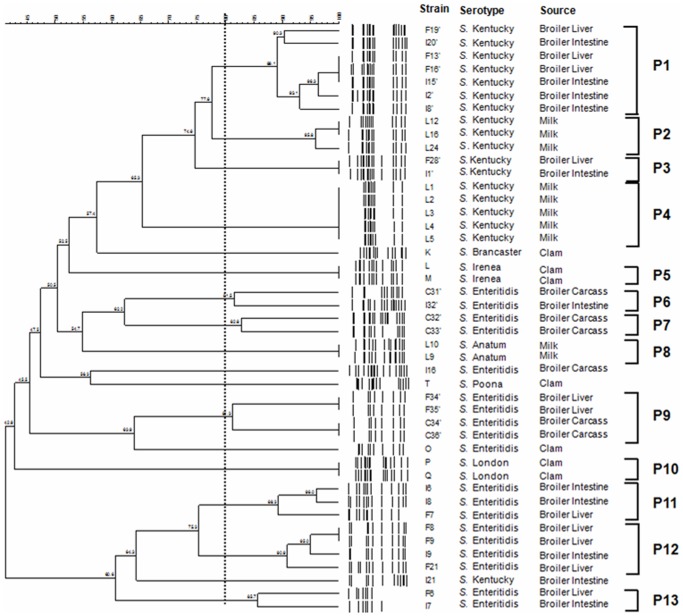
PFGE Dendrogram showing the relationship between *Salmonella* isolates. The similarities between strains were evaluated using the Dice coefficient and the UPGMA clustering method. Genetic similarity between samples in duplicate is 80%.

Our PFGE observations raise the question of whether *Salmonella* isolates from different food origins are phylogenetically related or comprise multiple lineages. Results show that most serovars, including *S.* Enteritidis and *S.* Kentucky, which are antigenically identical to each other, were assigned to multiple pulsotype profiles (Figure 1) and clusters (Figure 2). It is important to note that when three lambda markers were included on each gel, the genetic similarity between them was 80% (data not shown). Therefore we considered that two isolates presenting more than 80% of similarity were the same.

PFGE of XbaI-digested genomic DNA from 45 *Salmonella* isolates showed 13 different macrorestriction profiles or clusters (P1 to P13), while the remaining 5 isolates were unclustered. The latter belonged to serovars *S.* Enteritidis (I16 and O), *S.* Kentucky (I21), *S.* Poona (T) and *S.* Brancaster (K) (Figure 2). Six isolates were distributed among 3 clusters with two belonging to *S.* Irenea (P5), two isolates belonging to *S.* Anatum (P8) and two belonging to *S.* London (P10). Each cluster was composed of isolates derived from one origin, clam for P5 and P10 and milk for P8.

Heterogeneity was observed within the two major serovars, *S.* Enteritidis and *S.* Kentucky. The first serovar with 19 isolates was distributed among 2 unclustered isolates and 17 assigned to 6 PFGE clusters designated P6, P7, P9, P11, P12 and P13. About 95% of *Salmonella* isolates belonging to these clusters were derived from broiler samples (Figure 2) with 7 isolates from liver, 5 from intestine and 5 from total carcass.

The *S.* Kentucky serovar with 18 isolates was distributed among only one unclustered (I21) isolate and 17 assigned to 4 PFGE clusters designated P1, P2, P3 and P4 (Figure 2). Eight clustered isolates belonging to this serovar were derived from milk and nine from broiler samples (intestine or liver).

The discrimination index (DI) of PFGE in this analysis was found to be 0.94.

### ERIC-PCR typing analysis

**Figure 3:**
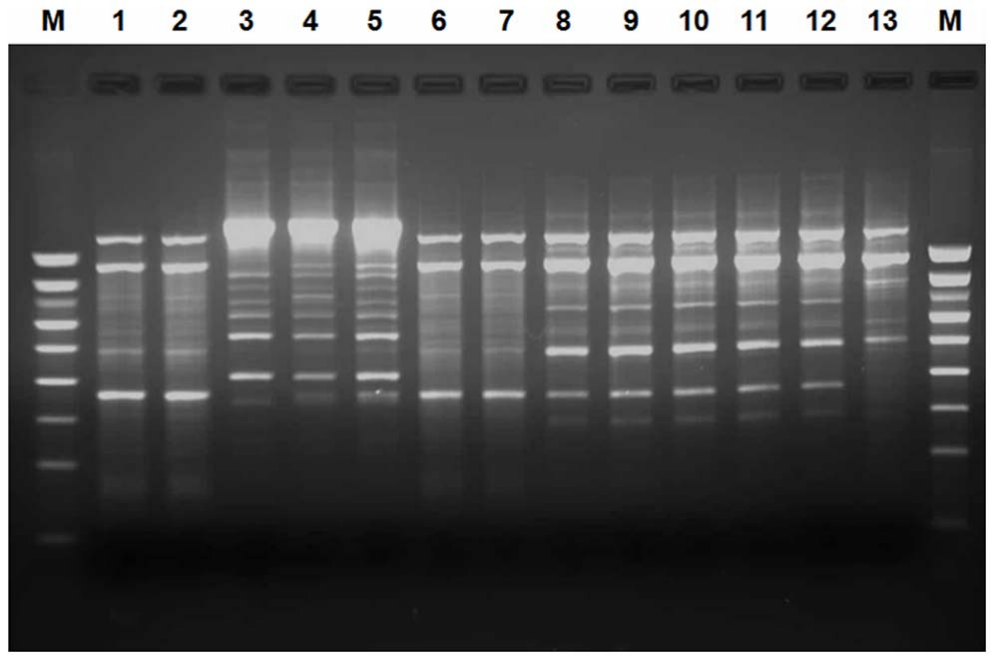
Representative ERIC-PCR fingerprint of different between *Salmonella* isolates on 2 per cent agarose gel. M: 1000 bp DNA marker; lane 1 to 13: F34′, F35′, C31′, C32′, C33′, C34′, C36′, L1, L2, L3, L4, L5 and L9.

**Figure 4:**
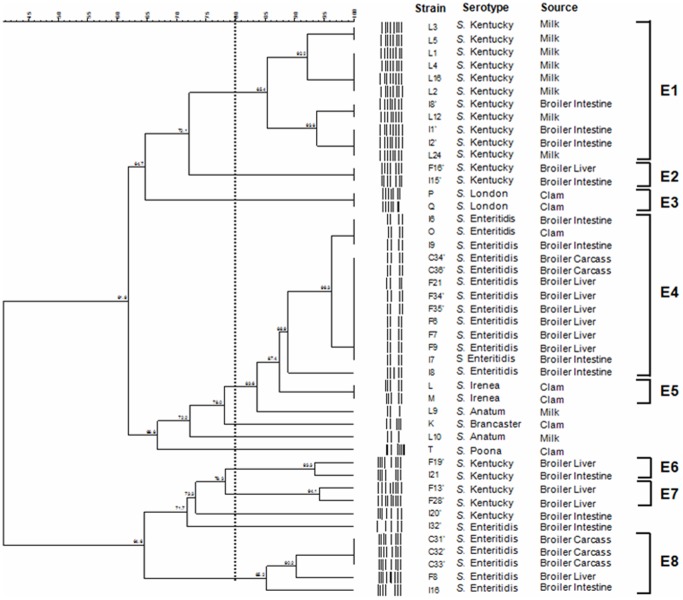
Genetic similarities of *Salmonella* strains isolated from clams, broilers and milk based on ERIC-PCR patterns. The dendrogram was generated by BioNumerics Software with the bande-matching coefficient of Dice and the UPGMA clustering. Genetic similarity between samples in duplicate is 80%.

Two 1000 base pair Smart Ladders were included on the gel as a marker. The genetic similarity between them was 80% (data not shown). The ERIC-PCR of 45 *Salmonella* isolates yielded different patterns consisting of 3–9 bands (Figure 3). All the serovars were grouped into 8 clusters (E1 to E8) while the remaining 6 isolates were unclustered. The ungrouped isolates belonged to serovars *S.* Enteritidis (I32′), *S.* Kentucky (I20′), *S.* Poona (T), *S.* Brancaster (K) and *S.* Anatum (L9 and L10) (Figure 4). Clustering based on fragment profiles grouped *S*. Enteritidis serovar into only two clusters (E4 and E8) (Figure 4). The second major group, *S.* Kentucky serovar, was distributed among only one unclustered isolate and 17 assigned to 4 ERIC clusters designated E1, E2, E6 and E7 (Figure 4).

Finally, *S.* Irenea (E5) and *S.* London (E3) clusters contained two *Salmonella* isolates each. The discrimination index (DI) of ERIC-PCR typing in this analysis was found to be 0.85.

### Composite analysis of PFGE and ERIC-PCR

**Figure 5:**
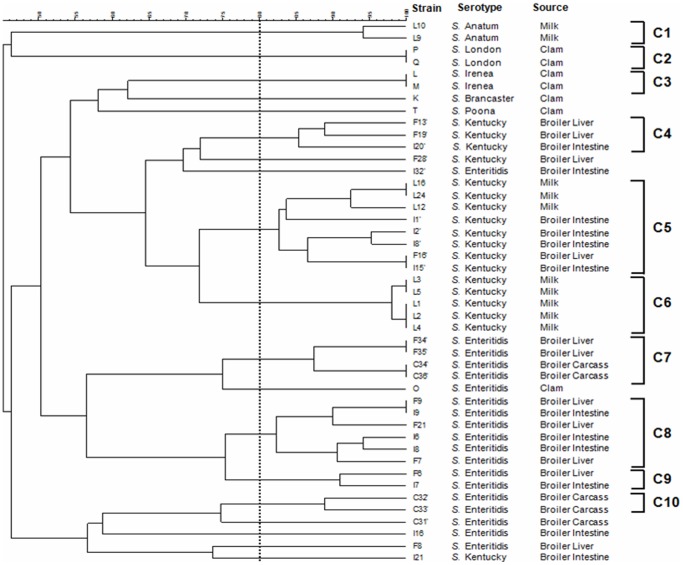
Dendrogram showing the percentage of similarity between typable *Salmonella* isolates generated from composite fingerprinting. Genetic similarity between samples in duplicate is 80%.

Data from the two molecular typing methods were subjected to a composite analysis to determine whether a better clustering of the serovars could be obtained. Clustering based on fragment profiles grouped the serovars into 10 clusters (C1–C10) (Figure 5). The 19 isolates of *S.* Enteritidis were grouped into 4 clusters (C7, C8, C9 and C10), while the remaining 5 isolates were distinct from each other. Except I21, all isolates belonging to S. Kentucky serovar were differentiated into 3 groups (C4, C5 and C6). *S.* Anatum (C1), *S.* London (C2) and *S.* Irenea (C3) were still grouped into two isolates per cluster. The combined PFGE-ERIC-PCR patterns allowed a DI of 0.93.

## Discussion

Salmonellosis is one of the most common causes of foodborne infection worldwide. *Salmonella spp*. can be isolated from different origins such as raw meat and poultry products as well as milk and milk based products [23].

This work focuses on the assessment of two molecular methods (PFGE and ERIC-PCR) for inter and intraserovar strains differentiation. These techniques were evaluated both alone and in combination for typing *Salmonella* isolates. The methods applied in our study are among those methods used for epidemiologic analysis of important zoonotic bacterial pathogens [24].

*Salmonella enterica* isolates isolated in this work have avian (broiler), sea products (clam) and bovine (milk) sources. All over the world, the most often isolated serovar is *S*. Enteritidis [25]. However, in this study, 42% versus 40% of the 45 isolates belonged to *S.* Enteritidis and *S.* Kentucky serovars, respectively, suggesting the emergence of *S.* Kentucky serovar in Tunisia.

All isolated strains were serotyped and confirmed as *Salmonella* strains using *invA* gene. The invasion gene *invA* is essential for full virulence in *Salmonella* and it is thought to trigger the internalization required for the invasion of deeper tissues [22].

The PFGE of XbaI-digested genomic DNA from 45 *Salmonella* isolates showed 13 different macrorestriction profiles or clusters (P1 to P13). Heterogeneity was mainly observed within the two major serovars, *S.* Enteritidis and *S.* Kentucky. The distribution of isolates in clusters was done independently of the origin of the broiler samples. In this study, only one isolate belonging to *S.* Enteritidis serovar (O) was obtained from clam and no one from milk. In an earlier investigation, researchers showed that among the 58 seafood associated *Salmonella* serovars, nine were observed but no one belonged to *S.* Enteritidis serovar [2]. However, *Salmonella Enteritidis* serovar has already been reported to survive and grow in fermented milks [26]–[27].

It is important to note that using the PFGE molecular typing method, heterogeneity was observed within *salmonella* belonging to the same serovar. Serological relatedness did not show correlation with genetic relatedness as previously reported in many studies [28]–[30]. The discrimination index (DI) of PFGE in this analysis was found to be 0.94. This typing method presented so that a very high discriminatory power (if complying with the conventional 5% level of acceptable probability where a DI>0.95 is desired [31].

The ERIC-PCR of the 45 *Salmonella* isolates also yielded different patterns grouped into 8 clusters (E1 to E8). In this case, the isolates distribution in clusters was done independently of the origin of the broiler samples.

Inter-serovars heterogeneity was observed even within the two *S.* Anatum isolates which were clustered in the PFGE dendrogram. This result proves the effectiveness of this molecular typing method and shows that ERIC-PCR could be useful for subtyping *Salmonella* serovars, where ubiquitous and similar PFGE patterns occur. Similar results were previously reported in many studies when ERIC-PCR was compared to other molecular typing methods [10]–[12]. Besides, the accuracy, simplicity and lower cost of ERIC PCR compared to other typing methods enhance its usefulness for *Salmonella* serovars analysis and it has been successfully used for typing many entero-bacteria [32]–[34].

Recently, a collection of 57 *Salmonella* Kentucky isolates was analyzed by Turki and others using plasmid profiling, PFGE, ribotyping, ERIC-PCR fingerprinting, and Random Amplification of Polymorphic DNA [35]. The authors showed a discriminatory index of 0.647 for PFGE versus 0.903 for ERIC-PCR. However, in the present work, results show that PFGE is more discriminative than ERIC-PCR to differentiate even intraserovars isolates. In fact, the DI of ERIC-PCR analysis was found to be 0.85. As regards discriminatory power alone, this datum shows that the DI obtained by ERIC-PCR is slightly below the acceptable confidence value for interpreting the discrimination level. Thus, PFGE typing method (with a DI of 0.94) is more discriminatory than ERIC-PCR which is insufficient in this case as a single typing method. Our result is in agreement with that of other workers who reported that PFGE is one of the most reliable techniques for discriminating different serotypes of *Salmonella* [29], [36].

Turki and others also indicate that a single method cannot be relied upon for discriminating between *S*. Kentucky strains, and a combination of typing methods such ERIC2 and RAPD2 allows further discrimination [35]. Data from the two molecular typing methods used in the present report were subjected to a composite analysis to determine whether a better serovars clustering could be obtained. The combined PFGE-ERIC-PCR patterns allowed a DI of 0.93 close to that obtained by PFGE and higher than that obtained by ERIC-PCR. In statistical terms, this would provide 93% confidence in the ability to accurately discriminate between two unrelated strains. Results of the combined analysis were highly discriminatory and thus more efficient as reported by Shariat et al. [30].

In the present work, the *Salmonella* search was positive in broiler intestine, liver and carcass. During the slaughter, this pathogen can contaminate carcasses and meat, resulting in a source of food borne illness [37]. Results show different profile patterns between 3 isolates derived from broiler carcass (C31′, C32′ and C33′) suggesting that a vertical transfer could occur. Bacterial contamination can be then spread to millions of chicks within few days via horizontal transfer. On the other hand, *S.* Kentucky isolates belonging to cluster C5 (Figure 5) derived from two different food borne origins and presented the same profile pattern. Finally, isolates derived from clams were always different from those derived from broiler and/or milk which is a further argument in favor of the diversity of *Salmonella* strains contaminating foods.

## Conclusions

In the current work, PFGE and ERIC-PCR were used for subtyping *Salmonella* isolates belonging to different serovars. An analysis of the two typing methods indicated that some of the *Salmonella* isolates were indistinguishable and/or highly related. These isolates were thus grouped into clusters whose number is higher when PFGE typing method was used. The dendrograms showed that PFGE and ERIC-PCR differentiated isolates into grouping that correlated with serovars. A wide variety of profiles was observed between the different serovars. This diversity is particularly important when the sample origins are varied, and even within the same sampling origin.
